# Developing integrated workflows for the digitisation of herbarium specimens using a modular and scalable approach

**DOI:** 10.3897/zookeys.209.3121

**Published:** 2012-07-20

**Authors:** Elspeth Haston, Robert Cubey, Martin Pullan, Hannah Atkins, David J Harris

**Affiliations:** 1 Royal Botanic Garden Edinburgh, 20a Inverleith Row, Edinburgh, EH3 5LR, UK

**Keywords:** Large-scale digitisation, curation, data entry, image capture

## Abstract

Digitisation programmes in many institutes frequently involve disparate and irregular funding, diverse selection criteria and scope, with different members of staff managing and operating the processes. These factors have influenced the decision at the Royal Botanic Garden Edinburgh to develop an integrated workflow for the digitisation of herbarium specimens which is modular and scalable to enable a single overall workflow to be used for all digitisation projects. This integrated workflow is comprised of three principal elements: a specimen workflow, a data workflow and an image workflow.

The specimen workflow is strongly linked to curatorial processes which will impact on the prioritisation, selection and preparation of the specimens. The importance of including a conservation element within the digitisation workflow is highlighted. The data workflow includes the concept of three main categories of collection data: label data, curatorial data and supplementary data. It is shown that each category of data has its own properties which influence the timing of data capture within the workflow. Development of software has been carried out for the rapid capture of curatorial data, and optical character recognition (OCR) software is being used to increase the efficiency of capturing label data and supplementary data. The large number and size of the images has necessitated the inclusion of automated systems within the image workflow.

## Introduction

The need for the digitisation of biological collections is widely recognised (eg European Commission 2011, Kroes 2011, Niggeman et al. 2011) resulting in the development of national digitisation strategies (eg Beach et al. 2010). The challenges of digitising natural history specimens have been explored (eg Beaman et al. 2007, Vollmar et al. 2010) and there have been several studies investigating data capture methods (Beaman et al. 2006, Heidorn and Wei 2008, Best et al. 2009, Lafferty and Landrum 2009, Granzow-de la Cerda and Beach 2010, Haston et al. 2012). Within this context of large scale digitisation of natural history collections, there is a need for the development of digitisation workflows to manage each of the elements of the digitisation process.

In developing workflows for the digitisation of herbarium specimens there are many factors which will influence the decisions made. Whilst it is clear that the financial costs of a digitisation programme may significantly limit the options available for equipment, software, staffing and storage, there are also other factors to consider. The funding itself may be irregular and be used for a range of diverse projects. Each institute has their own priorities and constraints and in the larger institutes there may be a range of digitisation programmes each with a different focus but which need to be integrated in some way. The recommendation of following a demand-driven digitisation model (Berendsohn and Seltmann 2010, Berendsohn et al. 2010, Berents et al. 2010) may result in an increase in the diversity of material being prioritised which will have an impact on the efficiency of the workflow. The concept of scalability is a factor which takes into account the potential increase in funding and resources. In addition, the integration of digitisation workflows into the core curation activities may play a large part in the decision-making process.

At the Royal Botanic Garden Edinburgh (RBGE), we have aimed to develop workflows which incorporate automated systems to enable us to expand and speed up the digitisation process. However, given the irregular nature of much of the funding available for digitisation, we have also based the digitisation workflows on a modular system which has the potential to be scaled up as funding becomes available. Additional modules may be added as they are developed, including a georeferencing tool (Llewellyn 2011) and additional quality control elements. A key factor in developing the workflow has been the need to continue to manage the images and data after capture. This is a very significant addition to the workload for herbarium staff and there is a requirement for this aspect of the workflow to be as efficient and simple as possible, with the aim of helping curators in the future to manage the collections.

Where possible, the digitisation workflow aims to use shared standards and formats. The adoption of standards allows easier transfer and sharing of data and is recognised as being of high importance in digitisation strategies (eg Beach et al. 2010). All data are routinely submitted to the Global Biodiversity Information Facility (GBIF), images are available on Encyclopedia of Life (EOL), a proportion of images and data are submitted to JSTOR and we are working on processes for submitting images and data to the Barcode of Life Database (BOLD) and Europeana.

## Workflows and processes

The integrated digitisation workflow at RBGE has been developed over the last four years, during which time it has evolved into the present system. Over this period large digitisation projects have been undertaken wholly within this system, whilst other projects have gradually been incorporated. All digitisation is now undertaken within this integrated system and there are currently 160,000 specimens digitised and available online (www.rbge.org.uk). Whilst increasing the rate of digitisation has been a contributing factor in the development of workflows, the need for managing the data, images and processes has been the most important driver for the development of this integrated system.

**Figure 1.**
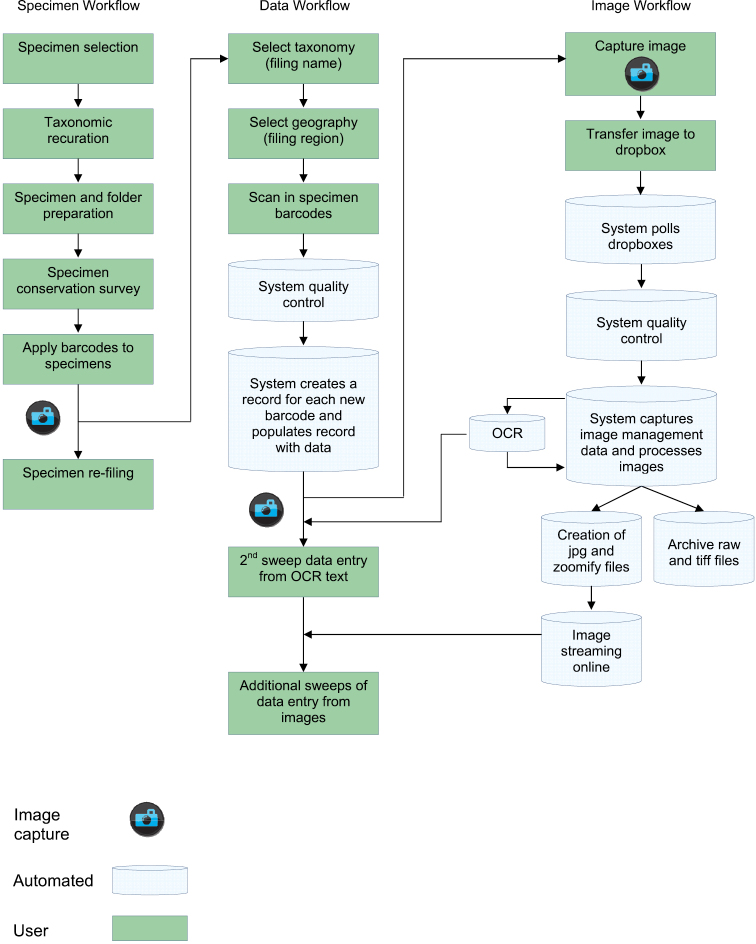
Diagrammatic overview of the digitisation workflows at the Royal Botanic Garden Edinburgh (RBGE)

There are three primary workflows within the digitisation programme (Fig. 1). The specimen workflow involves the physical movement and preparation of the specimens and folders. The data workflow focuses on the capture and management of specimen data (included within a broad “metadata” concept by Berendsohn et al. (2010)). Finally the image workflow focuses on the capture and management of images and related image management data including the equipment, operator and file location. These workflows and the interactions between them are described here.

### The specimen workflow

In this context, the specimen workflow involves the physical selection and movement of specimens within the digitisation process as well as the preparation of the specimens and folders. This is closely linked with existing specimen workflows for loans, incoming specimens, destructive sampling, curation etc.

See the Specimen workflow in Figure 1.

The selection of the specimens is dependent on the outcome of the prioritisation procedure. The specimen workflow developed at RBGE predominantly focuses on large taxonomic or geographical groups to increase efficiency, and scaling up small user requests to more manageable units based on taxonomy and geography. The prioritisation of specimens within the digitisation programme has been mainly influenced by RBGE research strategy as well as external projects. This has resulted in the selection of floristic areas such as SW Asia and the Middle East, as well as focus taxonomic groups such as Sapotaceae, Zingiberaceae, Begoniaceae and Gesneriaceae. Funding from the Andrew W Mellon Foundation through the Global Plants Initiative enabled us to digitise all the type specimens, which form another significant part of the collections.

The preparation element (ie taxonomic recuration and specimen & folder preparation) of the specimen workflow is an important factor which is often under-estimated. This fundamental curatorial work includes ensuring that the specimens are correctly filed and that the filing name is legible and clearly visible, as well as ensuring that the condition of the specimens is assessed and conservation work carried out as required.

The decision to keep the herbarium open and to maintain full access to the specimens as much as possible during the digitisation programme has been necessary due to the expected duration of the digitisation work given the current funding. In practice, this has resulted in the specimen workflow for digitisation being affected by many curatorial and research activities. We have therefore aimed to integrate the workflow and other curatorial workflows currently in place. An outcome of this integration has been the modification of some curatorial practices, including loan and destructive sampling procedures.

The digitisation workstations are currently all within the herbarium area to reduce the amount of movement and to remove the need for freezing specimens on return for pest control. We have aimed to keep the number of specimens out of the cabinets at any time to a minimum whilst working with a large enough unit to be efficient.

The inclusion of an assessment of the condition of the specimens and some preservation work has reduced the rate of digitisation. However, this work is critical for the conservation of the collections and incorporating this work within the digitisation programme when the specimens are being handled is allowing us to improve the condition of the specimens. The assessment of specimen condition can also be collated and used to inform strategic decisions about the overall management of the collections.

The scope of an individual digitisation project and the arrangement of the specimens within a herbarium has a large impact on the efficiency of the specimen workflow. Whilst the most efficient workflow would generally be to work through the collections cabinet by cabinet, this can be difficult to reconcile with digitisation projects based on a particular collector or country, or with demand-driven digitisation.

### The data workflow

The data workflow here includes all elements of capturing and managing data associated with the specimens, and linking these to the images and image management data. Logistically, the data associated with biological collections can be divided into three main categories for digitisation (Haston et al. 2012). Label data which are present on the specimen; curatorial data which are found on the containers holding the specimens; and supplementary data which are held separately from the collections in indexes, archives and literature. These data types can be captured using different methods at different stages of the data workflow.

Curatorial data are held separately from the specimen within the collections. At the Royal Botanic Garden Edinburgh this generally consists of two pieces of data: the filing name of the specimen and the broad geographical region from where it was collected. These represent the classification and location of the specimen within the collections, providing key information for the physical location and arrangement of specimens. Some or all of these data may not be present on the specimen itself as label data. This property means that the most efficient way to capture this data is from within the collection using information on the folders.

Label data are physically associated with the specimen and are generally visible in the corresponding digital image. This property allows these data to be captured at a later stage in the overall digitisation workflow. At RBGE, there is a small number of labels that are obscured by plant material or capsules which are not routinely captured.

Supplementary data such as field notebooks, citations in literature and online resources including Genbank, are independent from the label and curatorial data but can be used to enrich them.

See Data workflow in Figure 1.

The data workflow at the Royal Botanic Garden Edinburgh starts with the capture of curatorial data. Software written in PHP has been developed in-house to provide a simple web-based interface for rapid capture of the filing name, geographical region and barcode assigned to each specimen. The interface is designed around the fact that at the lowest level specimen storage within the herbarium is arranged into separate folders for each species within a geographical region. Within the interface users can select the species and geographical region for the folder and then add the individual specimens in each folder simply by scanning the barcode on the specimen. A specimen record is created for each barcode scanned and cross checked against any existing records in the herbarium database. After validation and error correction the new records are then batch imported into the herbarium database (*BG-BASE*^TM^ version 6.8). A similar tool has now been developed within *BG-BASE*^TM^.

Once the specimen has been imaged, label data can be captured during subsequent sweeps of data entry. Specimen images are processed through optical character recognition (OCR) software (ABBYY Recognition Server v. 3.0). At present the resulting text is stored unparsed as a single data string. This is then searched for recognisable tags (characters) to allow the creation of subsets of images and specimen records. These subsets are visually checked to ensure the selection process was correct and then the relevant data automatically entered. This is currently being carried out for collector and country. Finally, additional sweeps of label data entry are carried out by operators using a combination of the images and OCR text.

Within a modular system a level of data entry can be independent from imaging. This allows the ability to tailor the work being undertaken to the resources available. The use of minimal data capture methods enables the rapid creation of placeholder records that give collection managers valuable information about the number of specimens within a taxon for a particular filing region, and thus act as a catalogue of the collections. These also act as placeholder records to which images and OCR data can be attached, and which can be expanded as and when additional resources and technology become available.

The overall workflow is designed to accommodate the different requirements of the separate projects being undertaken simultaneously in the herbarium. These requirements may vary from full data entry with an image as is usually required for taxonomic or floristic work, partial data with georeferenced locality which is often all that is required for biogeographic studies, through to a basic catalogue record with minimal data for curation purposes. All these requirements can be handled within the one system. This is of particular importance due to the irregular nature of funding.

### The image workflow

Digitisation projects are resulting in large numbers of high quality images of approximately 150MB each. The scale of the digitisation programmes is too large for completely manual processes to be used to manage these images. Image workflows being developed at the Royal Botanic Garden Edinburgh include image capture, processing, image management data recording, optical character recognition (OCR), quality control, image streaming online and archiving. This is carried out within a system based on image server software written in-house. This software has been written in Visual Basic and is designed to run as a Windows service. The software is responsible for marshalling newly scanned images into their ultimate destinations, registering the new images and all derived versions of that image in the image database, creating rescaled web viewable versions of each image, tiling the images to allow web presentation of zoomable versions of the image, submitting the images for OCR processing and recording the results of the OCR process in the image database. Multiple instances of the service can be installed in multiple servers to allow parallel processing of the new images.

See the Image workflow in Figure 1.

Image capture is carried out using two methods, but which feed into the same image workflow system. Epson Expression Model 10000XL scanners at 600dpi, result in tiff files of approximately 150–200MB each. The Leaf Aptus II-10 56 megapixel digital backs result in raw files of approximately 100MB from which tiffs of approximately 150MB are created using LeafCapture software. Preliminary quality control checks are carried out at this stage. These include manually checking the focus and cropping, as well as ensuring that the tiff file has been sucessfully created.

All images are then saved to a dropbox folder structure. The folder names comprise basic image management data including the equipment and operator’s name. The image server software continuously polls the dropboxes for new files.

Additional automated quality control checks are carried out at this stage to ensure that the files are within acceptable size boundaries, that the filename fits a standard pattern, and that an electronic file with the same filename does not already exist.

As they appear, the system records associated image management data, including the equipment and operator’s name, into an image database. The system then creates fully tiled image files which are stored in a zip compressed file. The tiles are extracted from the compressed file by the image server software in response to tile requests from the image viewer. The image viewer is an embedded object contained with the HTML page presented to a web browser. We currently use Zoomify image viewer software (Zoomify Enterprise ^TM^).

A jpg file of approximately 1MB is also created and made available online. A copy of the tiff file is transferred to the ABBYY OCR workflow (ABBYY Recognition Server 3.0). Finally, the raw and tiff files are saved to archive folders, to be stored offline on tape and external hard drive storage. The locations of all these files are recorded within the image database.

This system has been developed as a modular system which can be extended as the number of cameras or scanners increase. There has been an emphasis on developing more automated systems but which can allow an element of user interaction if required, particularly within the quality control elements.

The decision to capture the images at 600dpi or equivalent was based on producing images which contain the same visible information as would be available through the standard taxonomic tool of a 10× hand lens. This results in very large images in excess of 150MB which create significant image management problems. There is currently debate about the need for such high resolution and RBGE has been involved in these discussions. We have felt the need to maintain this high level of resolution to ensure that sufficient information is retained, and in the understanding that these images can be scaled down in the future but cannot be scaled up.

Deliberately keeping both the raw and the tiff formats increases the demands on storage. In the rapidly changing environment of image file formats we aim to be inclusive and retain our ability to adapt to future developments.

## Discussion

The workflows developed here have been strongly influenced by the presence of different funding streams and a diversity of digitisation projects along with the need to create a modular and integrated workflow to manage the processes, images and data. This is in contrast to an alternative digitisation approach such as that seen at the Muséum national d’histoire naturelle (MNHN) in Paris which aims to digitise all specimens within a single project, using a more unified approach.

A single unified structure may reduce the problems inherent in a modular system in which linking and maintaining links between software developed by different programmers and residing on different servers can be an issue as versions change over time. In contrast, a modular approach can potentially benefit more readily from advances in technology as modules can be added, updated or replaced as they become available.

One of the benefits of the modular system developed at RBGE has been to create an integrated but flexible management structure for specimens, data and images, which reduces the need for individual projects to create their own systems with the additional time and costs involved.

A second and highly significant benefit of an integrated system is that it helps with the curation of images and data post capture. It is essential that the on-going curation of these digital collections is considered as early as possible. The data and images need to be available and accessible but they also need to be kept up to date (with new determinations and additional data) and new file and archive formats. Having data in multiple systems, managed by different projects makes this on-going curation task almost impossible. Having them in one system makes this daunting and ever-growing task more achievable.
